# Prevalence and activity of class II microcins in *Serratia marcescens* strains by isolation source

**DOI:** 10.1128/aem.00259-26

**Published:** 2026-03-31

**Authors:** Jennifer K. Parker, Leon P. Toursarkissian, Joanna R. Chang, Simon Sanchez-Paiva, Angela C. O'Donnell, Halimot O. Badmus, Maria Patricia Nunez, Anne-Catrin Uhlemann, Bryan W. Davies

**Affiliations:** 1 Department of Molecular Biosciences, The University of Texas at Austin 196204, Austin, Texas, USA; 2 Department of Microbiology & Immunology, Columbia University Irving Medical Center, New York, New York, USA; 3 Division of Infectious Diseases, Columbia University Irving Medical Center, New York, New York, USA; 4 John Ring LaMontagne Center for Infectious Diseases, The University of Texas at Austin 12330, Austin, Texas, USA; Indiana University Bloomington, Bloomington, Indiana, USA

**Keywords:** antibacterial, competition, bacteriocin, secretion, gram-negative, microcin

## Abstract

*Serratia marcescens* is a highly environmentally adaptable gram-negative bacterium which can be found in both free-living and host-associated lifestyles, where it can function as either a commensal or a pathogen. The diverse array of secreted factors produced by *S. marcescens* is suggested to facilitate this adaptability. Here, we describe and validate a class of secreted bacteriocins, called class II microcins, which have not previously been described in *S. marcescens*. Putative class II microcins are found in the majority (96%) of genomes analyzed here, including representative strains from numerous diverse lifestyles. The type of microcin in a given genome has some correspondence to the isolation source of its *S. marcescens* strain of origin. We demonstrate that heterologous secretion of these microcins can be used to validate their antibacterial activity, yielding 17 confirmed class II microcins from *S. marcescens*. Testing against a panel of *S. marcescens*, consisting predominantly of strains pathogenic to humans or insects, shows *S. marcescens* microcin activity ranges from narrow to broad spectrum. Identifying class II microcins and other factors which may contribute to interbacterial competition or colonization by *S. marcescens* could inform strategies to prevent *S. marcescens* infections.

**IMPORTANCE:**

*Serratia marcescens* is an abundant bacterium in many different environments, but it also represents an important opportunistic pathogen of humans and other animals. Our finding that *S. marcescens* encodes numerous antibacterial class II microcins is important to understanding factors that may contribute to the lifestyle versatility of this bacterium. Interfering with factors that promote competition or colonization could aid infection prevention.

## INTRODUCTION

Class II microcins are a group of small bacteriocins which have long been known to be produced by a few species of *Enterobacteriaceae*, primarily *E. coli*. Some class II microcins have shown promise as regulators of pathogens within the gut microbiome (1–3), generating interest in microcins as tools to control specific bacteria or regulate the microbiome (4). Recently, a proliferation of evidence shows that class II microcins have unaccounted for diversity and abundance within and beyond the *Enterobacteriaceae* (5, 6), including functionally characterized examples from *Acinetobacter baumanii* (*Moraxellaceae*) (7), *Vibrio cholerae* (*Vibrionaceae*) (8), and numerous species of *Enterobacteriaceae* (9).

Our previous systematic *in silico* evaluation of class II microcin prevalence in a large collection of *E. coli* genomes found that 23.5% of genome assemblies contained confirmed or putative microcins (5). However, a superficial scan of select non-*Enterobacteriaceae* genomes (one representative genome per species) suggested far greater prevalence in the *Yersiniaceae*, at 51.9% of genomes (5). Closer inspection indicated that *Serratia* spp. accounted for much of this prevalence; 80% of *Serratia* spp. genomes in this small data set contained putative class II microcins. The most well-known species, *Serratia marcescens*, inhabits an array of aquatic, soil, and host organism environments and possesses a highly plastic genome (10). Importantly, it is an opportunistic pathogen of diverse unrelated taxa including humans (11), honey bees (12), and corals (13), making finding new options to regulate or treat *S. marcescens* infections a valuable endeavor.

Here, we describe the diversity and prevalence of *S. marcescens* class II microcins and confirm their antibacterial activity. Examination of a large collection of *S. marcescens* genomes with our bioinformatic pipeline (9) reveals that microcins are present in the majority of strains from this species, usually with multiple microcins found within a single genome. Furthermore, we demonstrate that some of these secreted microcins have antibacterial activity against *S. marcescens*, including multidrug-resistant strains, and that microcin type has some correspondence to the environmental isolation source of its strain of origin. This broad prevalence suggests that class II microcins convey some ecological advantage for *S. marcescens* that remains to be examined.

## RESULTS

### Putative class II microcins are highly abundant in *Serratia marcescens* genomes

Using the first iteration of our class II microcin detection pipeline (5), 1,621 *S. marcescens* genome assemblies were screened *in silico* for class II microcins, yielding 2,175 hits from 1,508 (93%) of the assemblies (File S1). We proceeded with only those hits that terminated in a stop codon (*n* = 2,153) and deduplicated the sequences, resulting in a total of 93 unique hits (File S1). These unique putative microcins were named by their species of origin (*Serratia marcescens*) plus a chronological number: SM001-SM080. The remaining 13 unique sequences differed only in their cleavable signal sequence, rather than the core microcin sequence; these are named by appending “difsig” (different signal) to the name of the microcin with the same core sequence, e.g., SM017 and difsigSM017 have the same core sequence but a different signal sequence.

Class II microcins are divided into class IIa and class IIb (14). The C-termini of class IIb microcins contain a ~10 amino acid glycine- and serine-rich sequence motif (15). This motif is recognized by co-locally encoded post-translational modification proteins which attach a siderophore moiety to facilitate target cell uptake of the microcin (15). Notably, none of the 93 putative class II *S. marcescens* microcin sequences possess the canonical class IIb C-terminus motif (File S1). This suggests they are either class IIa microcins, which have no post-translational modifications other than disulfide bonds (14), or an as-yet-defined subclass of microcins.

A phylogeny of the 93 unique sequences (Fig. S1) was generated to guide selection of putative class II microcins for antibacterial activity screening. As with our previous phylogenetic analyses of class II microcins (5, 9), many basal relationships are unresolved, but some well-supported clades are observed. We labeled 11 major clades (clades I–XI), leaving 5 single, unrelated sequences. Among the 80 unique core microcin sequences, 40 abundant and/or diverse sequences were selected for cloning and testing (Fig. S1; File S1). Among these 40 selected sequences, 2 are described in our recent work on class II microcins in the *Enterobacteriaceae* (9). Here, sequences SM012 and SM070 identified in *S. marcescens* are identical to putative microcin EN313 and confirmed microcin EN663, respectively, which are encoded by *Klebsiella pneumoniae*. Antibacterial activity screening of these 40 putative class II microcins is described in further detail below.

**Fig 1:**
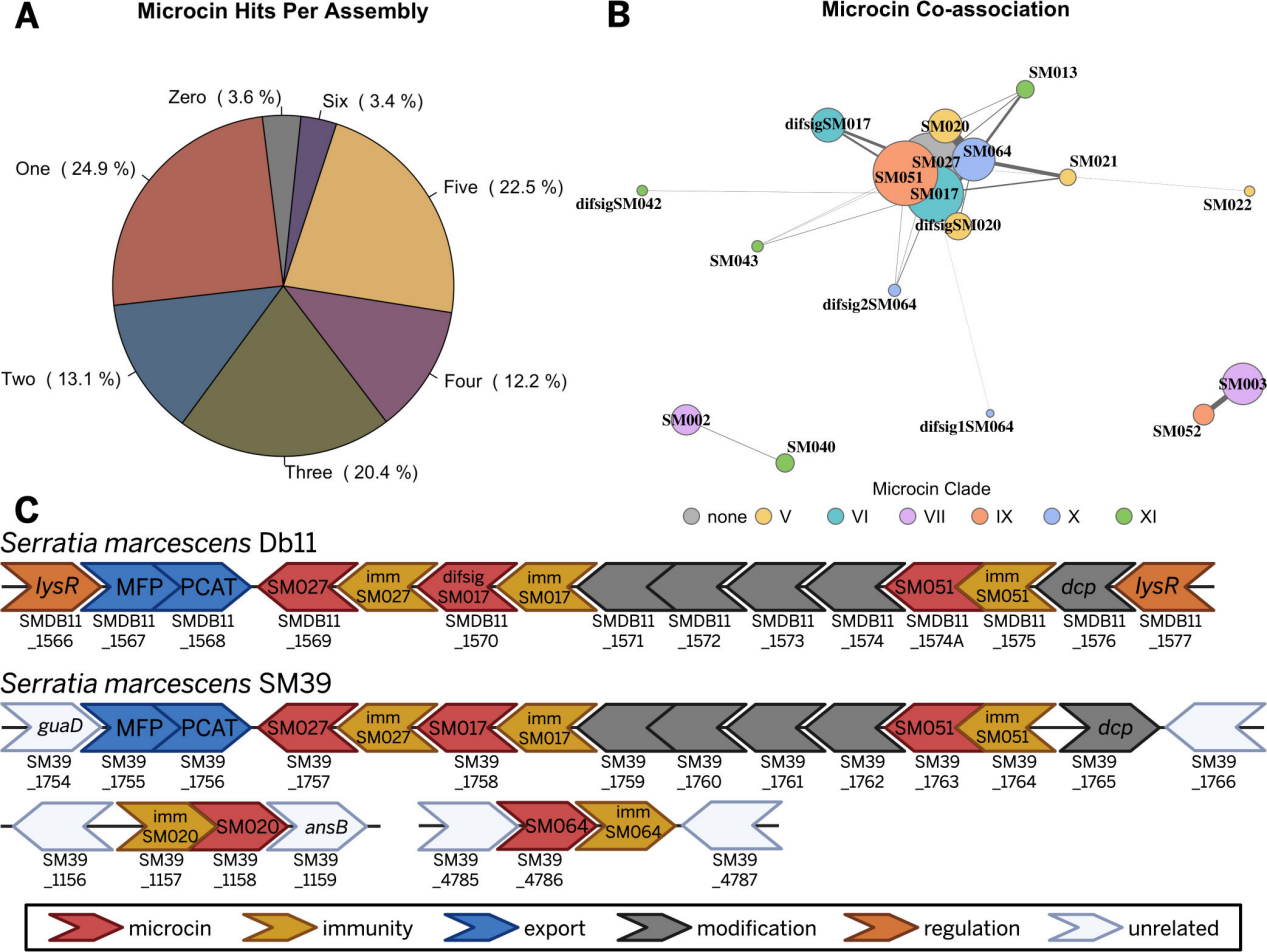
Class II microcin prevalence among *Serratia marcescens* strains. (**A**) Pie slices represent the proportion of *S. marcescens* genome assemblies (*n* = 1,621) containing 0–6 of 93 unique putative microcin hits predicted *in silico*. The majority of strains (96%) encode at least one putative class II microcin. (**B**) Co-association of different putative class II microcins within the same *S. marcescens* genome. A co-association network plot was generated for the 18 microcins found to be co-associated with one or more other microcins from 1,562 genome assemblies. Edge widths represent the strength of the association between a microcin pair as determined by chi-square tests, as calculated using the negative logarithm of resulting *P*-values. Node size is proportional to the percentage of total hits, normalized by the square root, and nodes are colored by microcin clades identified from phylogenetic analysis. (**C**) Example gene organization for co-associated putative microcins SM027, difsigSM017/SM017, and SM051 from *S. marcescens* isolates Db11 and SM39. Isolate SM39 also encodes putative microcins SM020 and SM064 in different genomic locations. Loci, with locus tags listed beneath if annotated on the genome assembly, are colored by putative function.

While the present work was completed using our original class II microcin detection pipeline (5) as described above, our subsequent publication of an updated pipeline (9) provided the opportunity to test its ability to improve genomic detection of the 93 unique putative microcins in our study data set and update their abundance data accordingly. We reanalyzed the genome assembly data set with the improved pipeline, which increased the total count of detected microcins; 4,809 hits to the 93 putative microcins were identified among 1,562 (96%) of the assemblies (average of 3 hits per assembly; File S1). Though some new sequences were identified (File S1), we restricted our downstream analyses to the 93 original sequences. Because their amino acid sequences are the same as those found by the first pipeline, new hits to these 93 putative microcins can be attributed to improved open-reading frame (ORF) detection, rather than changes to the profile hidden Markov model (pHMM) (9). Hits were dominated by a small number of highly abundant sequences: the top 13 most abundant sequences (all comprising >1% of total hits) accounted for 89.2% of hits (File S1). SM027, SM051, and SM017 were particularly prevalent; they were detected in 56%, 55%, and 43%, respectively, of all assemblies containing microcins. Multiple microcins were usually detected per assembly (Fig. 1A); of assemblies with putative microcins, 74% encoded more than one microcin.

Due to the prevalence of genomes containing multiple putative class II microcins, the 93 unique microcin sequences were assessed for co-association. A total of 18 unique microcin sequences were co-associated with another microcin in 31 combinations (File S1). A network plot (Fig. 1B) showed that their co-associations resolve into three separate clusters: one large group of 14 putative microcins and two pairs of co-associated microcins. The four most abundant putative microcins (SM027, SM051, SM017, and SM064) were strongly associated with each other in the largest cluster. Among all *S. marcescens* assemblies analyzed here, 372 (23%) encoded all four of these microcins.

To provide additional support for class II microcin production by *S. marcescens*, assemblies encoding putative microcins were screened for evidence of their secretion machinery. Class II microcins are secreted via a microcin type I secretion system (mT1SS) (16). A key component of the mT1SS is the peptidase-containing ABC transporter (PCAT), which is required to recognize and cleave the microcin signal sequence in the initial step of export (17–19). Among the 1,562 assemblies with putative microcins, all but 5 (0.3%) encoded a PCAT (File S1). While most encoded a single PCAT (52.6%), many encoded more than one (47.1%).

To examine the genetic organization of some high abundance, co-associated putative microcins, we selected *S. marcescens* Db11 and SM39 (10), an insect isolate (*Drosophila melanogaster*) and a human multidrug-resistant clinical isolate, respectively. Their complete genome sequences have been compared previously (10). Both strains encode SM027, difsigSM017/SM017, and SM051 in close proximity to each other (Fig. 1C). Adjacent, but encoded in the opposite direction, are the PCAT and membrane fusion protein (MFP) (20) needed for export. Encoded among the microcins, there are several potential post-translational modification genes with BLAST similarity to the following: non-ribosomal peptide synthetase/amino acid adenylation domain-containing protein, AMP-binding protein, toxin-activating lysine-acyltransferase, MchC protein (modification protein for MccH47) (21), and Dcp (dipeptidyl carboxypeptidase II). Though the putative *S. marcescens* microcins lack an obvious canonical class IIb C-terminal sequence motif (4), in this example (Fig. 1C), SM051 has abundant C-terminal serines and is nearest of the three putative microcins to the potential modification genes. *S. marcescens* SM39 also encodes SM020 and SM064, but these are encoded at different locations in the genome (Fig. 1C). Most potential microcin immunity proteins are unannotated, consistent with our previous findings that immunity proteins, which are often small, can be overlooked by annotation software and may need to be manually predicted (9).

### *E. coli* microcin type I secretion system is configured to secrete *S. marcescens* microcins

We previously demonstrated the use of an *E. coli* mT1SS for heterologous secretion of class II microcins and other small proteins directly to the extracellular medium (8, 9, 22). We planned to use this *E. coli* mT1SS for secretion of putative *S. marcescens* class II microcins to confirm their antibacterial activity against a panel of *S. marcescens* strains. Heterologous production from *E. coli* DH5α, a K-12 derivative strain, was selected to eliminate interference from other natively produced *S. marcescens* antibacterials (23) and because factors regulating native microcin expression are poorly characterized (4).

First, it was necessary to determine if the microcins had a toxic effect on the *E. coli* secretor, which could inhibit their efficient secretion. *E. coli* and *S. marcescens* are members of the Enterobacterales order, albeit in different families (*Enterobacteriaceae* and *Yersiniaceae*, respectively) (24). Though microcins tend to be active toward species more closely related to their species of origin (14), they can also be active toward more distantly related species (9). So, while we hypothesized that some *S. marcescens* microcins would be active toward *S. marcescens* strains, we could not exclude activity toward other species, including *E. coli*, *a priori*.

**Fig 2:**
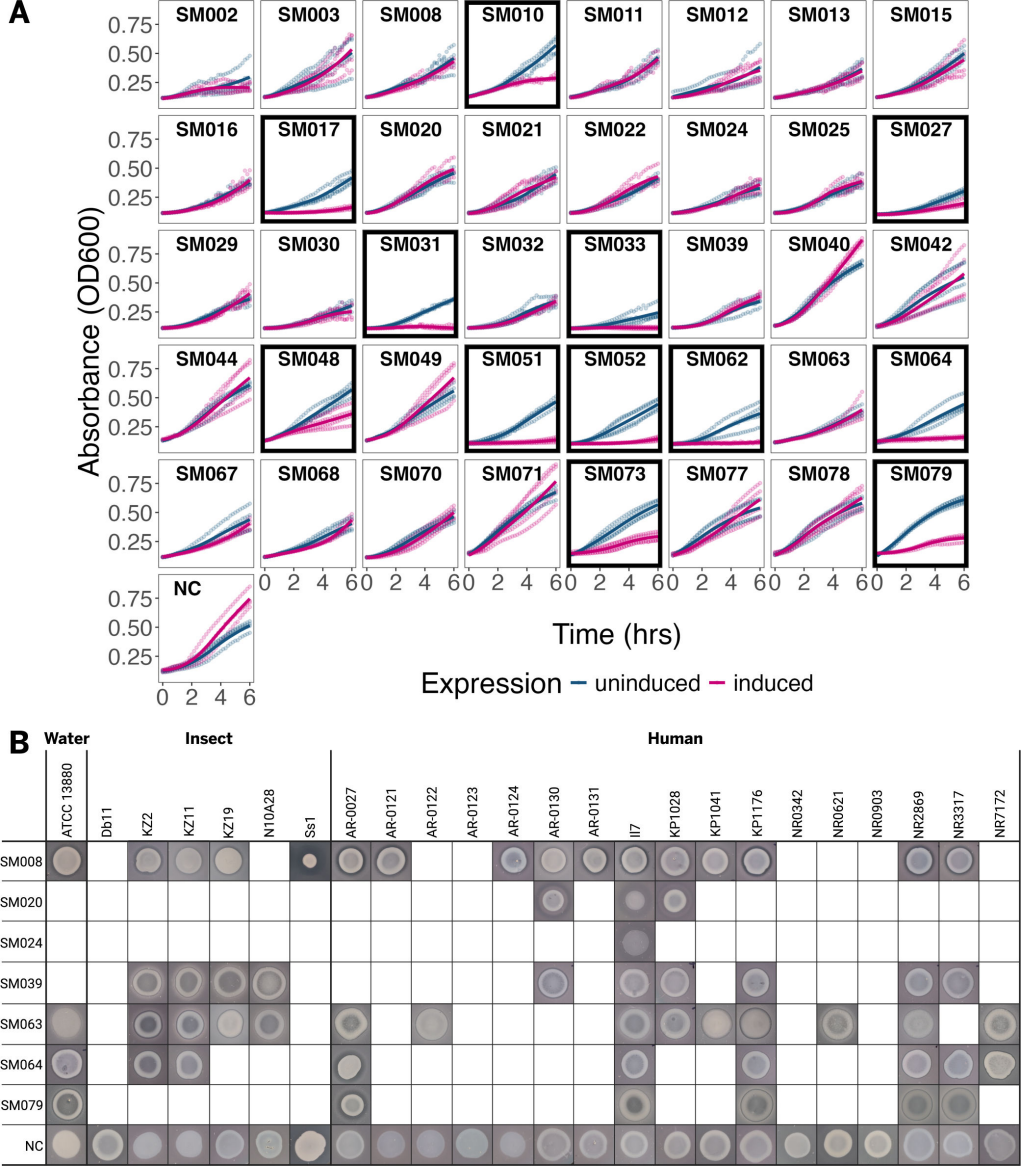
Antibacterial activity testing of putative class II microcins from *Serratia marcescens*. (**A**) Growth inhibition of *Escherichia coli* during heterologous secretion of *S. marcescens* microcins in the absence of a cognate immunity protein. Sequence diverse *S. marcescens* microcins (*n* = 40) and an empty vector negative control (NC) were heterologously secreted from *E. coli* via a microcin type I secretion system (mT1SS). Cell density (absorbance at OD600), with or without induction of microcin expression, was monitored for 6 h. A microcin is considered antibacterial (*n* = 12; plots in black boxes) if *E. coli* growth is significantly inhibited when microcin expression is induced compared to the uninduced control (*P* = 0.029, Wilcoxon rank-sum tests of area under the curve). Inhibition of the *E. coli* secretor suggests that a cognate microcin immunity protein is needed for efficient heterologous secretion. (**B**) Growth inhibition of *Serratia marcescens* by class II microcins. Class II microcins from panel A were tested for antibacterial activity against 24 strains of *S. marcescens* in zone of inhibition (ZOI) assays. Each *E. coli* secretor strain, coexpressing a microcin and its cognate immunity protein, was spotted onto an agar lawn containing an *S. marcescens* target strain and incubated to observe growth inhibition. ZOI are shown only for the 7 microcins (*y*-axis) which produced a ZOI on one or more of a panel of 24 strains of *S. marcescens* (*x*-axis). Results from an empty vector negative control (NC) secretor, with no microcin encoded, are also shown for all *S. marcescens* strains. Strains are grouped by isolation origin (water, insect, or human). ZOI images shown here are representative of an assay performed at least in triplicate.

The 40 *S. marcescens* class II microcins selected *in silico* (Fig. S1), and an empty vector negative control (NC), were cloned and transformed into *E. coli* containing the mT1SS. These *E. coli* strains were then assessed for growth inhibition during secretion of their respective putative microcins, as we have done previously (9). The optical densities (OD600) of *E. coli* strains in liquid culture were measured over time, with and without induction of microcin expression, and the area under the curve (AUC) was computed per growth curve. For 12 of the *E. coli* strains tested (Fig. 2A), growth was inhibited when microcin expression was induced compared to the uninduced condition (*P* = 0.029, Wilcoxon rank-sum tests), indicating that these microcins are antibacterial toward the secreting strain. This screening methodology was previously validated to detect antibacterial activity of class IIa microcins and some class IIb microcins (9).

For microcins which inhibited the growth of their *E. coli* secretor, we needed to identify an immunity protein. Class II microcins are natively encoded adjacent to their cognate immunity protein, which is canonically an inner membrane protein that protects the microcin-producing cell from microcin toxicity. Coexpression of the cognate immunity protein in our mT1SS can enable efficient microcin secretion and detection of antibacterial activity toward target strains of interest (9). Immunity proteins (File S1) were predicted *in silico* for all 12 microcins which inhibited the growth of the *E. coli* secretor (Fig. 2A) and for 9 select microcins which did not obviously inhibit *E. coli* growth, in case these also improved secretion.

### Secreted *S. marcescens* microcins have antibacterial activity toward *S. marcescens*

*E. coli* mT1SS strains secreting the 40 selected *S. marcescens* microcins, with and/or without putative cognate immunity proteins encoded and coexpressed (File S1), were tested for antibacterial activity against a panel of 24 *S. marcescens* isolates. These isolates originated from humans (*n* = 17), insects (*n* = 6), or water (*n* = 1; the species type strain). Human-origin isolates included both carbapenem-susceptible and carbapenem-resistant isolates; carbapenems are the last line treatment for complicated and/or multi-drug-resistant *Serratia* infections. A zone of inhibition (ZOI) assay was employed to test for microcin antibacterial activity, where the *E. coli* microcin secretor was spotted onto an agar plate containing a bacterial lawn of each target *S. marcescens* strain (Fig. 2B) (9). When microcin expression is induced, growth of a susceptible target strain is inhibited in the area around the secretor.

Initially, ZOIs from microcin secretors co-encoding putative immunity proteins (*n* = 21) were compared to ZOIs from the corresponding microcin-only secretors. If ZOI size/intensity was either unaffected or improved by coexpression of the putative cognate immunity protein, then the immunity-encoding strain was used in all final ZOI analyses. Immunity coexpression was selected for all seven microcins identified as antibacterial against *S. marcescens* based on these initial ZOI comparisons (Fig. S2).

Seven of the 40 class II microcins tested produced a ZOI on one or more of the 24 *S. marcescens* target strains under the conditions tested (Fig. 2B; File S2). The diameter and intensity of ZOIs produced by each microcin varied by strain. One or more active microcins were identified to inhibit 20 of the 24 target strains. SM008 is the most broadly active of the microcins tested here. Secreted SM008 inhibits the growth of 16 (67%) of the *S. marcescens* strains analyzed. Interestingly, the two broadest antibacterial range microcins (SM008 and SM063; Fig. 2B; File S2) were found rarely in the genome assembly data set: SM008 was found 19 times (0.40% of hits), and SM063 was found 3 times (0.06% of hits) (File S1).

Because 12 *S. marcescens* class II microcins inhibited the growth of the *E. coli* secretor in self-inhibition growth curve assays (Fig. 2A), we followed up with ZOI assays to determine if secreted *S. marcescens* microcins could also inhibit *E. coli* target cells. With co-encoded putative immunity proteins now available to ostensibly protect the *E. coli* secretor strains, ZOI assays were conducted against *E. coli* W3110, a K-12 derivative closely related to the DH5α secretor (25). No ZOIs against *E. coli* W3110 were observed (Fig. S3A). To explain this difference, we considered the possibility that a portion of the microcin exported from the secretion system is leaked into the periplasm, and this causes *E. coli* self-inhibition (Fig. 2A), rather than re-entry of the fully exported microcin across the outer membrane. Mature microcins contain a C-terminal uptake domain (interacts with the outer membrane receptor) and an N-terminal antibacterial domain (interacts with the inner membrane receptor/target) (26). If a strain is susceptible to a microcin’s antibacterial domain, but not its uptake domain, periplasmic leakage of secreted microcin would result in self-inhibition, but exogenous microcin would not produce a ZOI. Periplasmic leakage during secretion has been reported for MccV (27), which we show here using a split-luciferase complementation assay (28) (Fig. S3B and C); this leakage may explain why *E. coli* self-inhibition is seen during growth curves, but there is no evidence of direct inhibition during ZOI assays. Nonetheless, including both the *S. marcescens* ZOI results presented here (Fig. 2B; Fig. S2), as well as evidence from *E. coli* self-inhibition growth curves (Fig. 2A), we have demonstrated antibacterial activity for 17 microcins from *S. marcescens*.

### Relationships between microcin sequence, antibacterial activity, and isolation source

**Fig 3:**
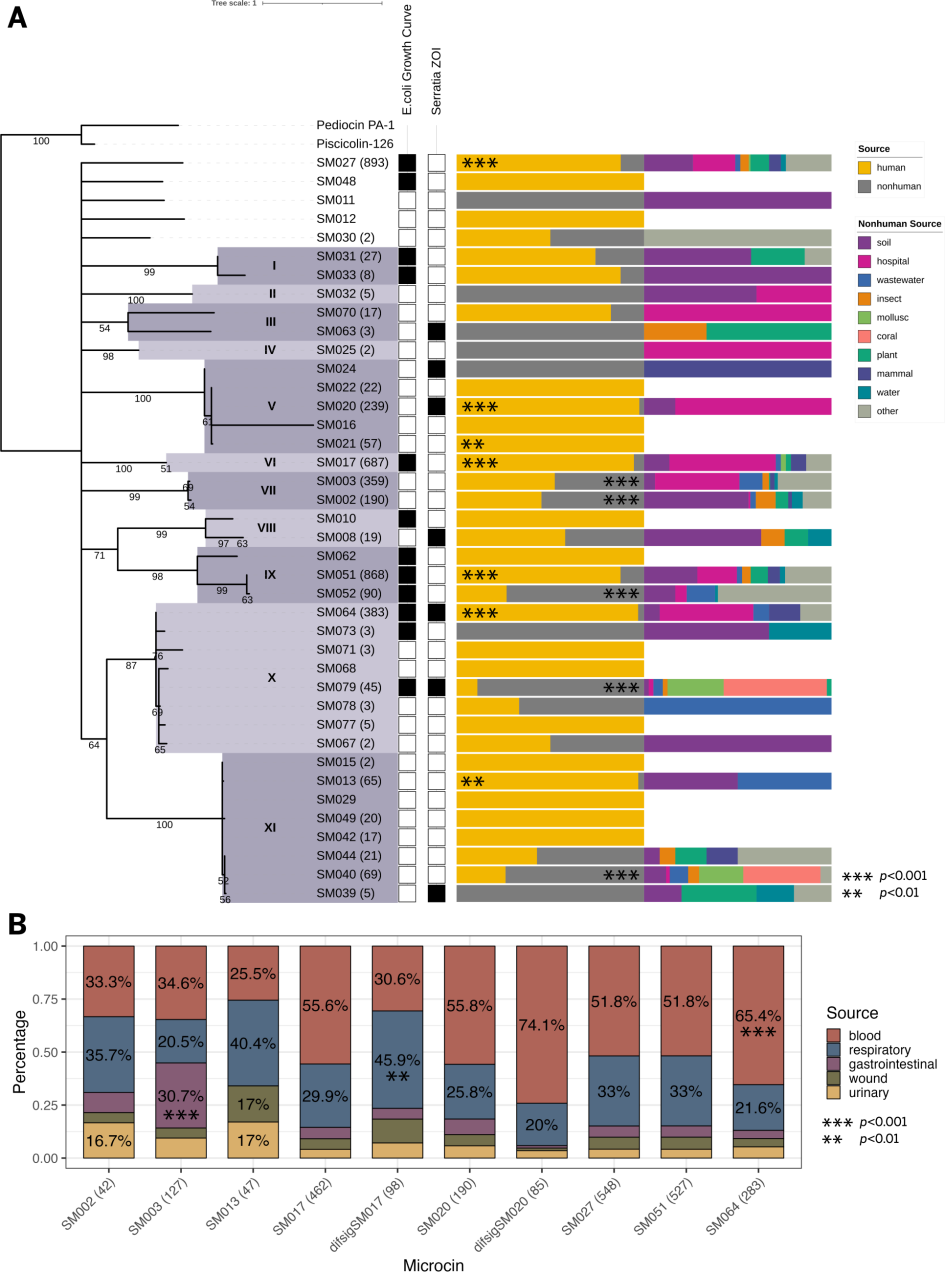
Relationships between *Serratia marcescens* microcin phylogenetics, antibacterial activity, and source. (**A**) A phylogeny of 93 unique putative *S. marcescens* microcin sequences was pruned to display the 40 sequences tested for antibacterial activity. Major clades of microcins are indicated by roman numerals; clade designations preserve the structure of the unpruned phylogeny (Fig. S1). Each microcin ID in the phylogeny is followed by the number of observed hits among the 1,621 screened genome assemblies in parentheses, if greater than one. Per microcin, presence/absence of antibacterial activity detected in two different assays is indicated. “*E. coli* growth curve” reflects the detection of growth inhibition of the *E. coli* microcin secretor in a growth curve assay. “*Serratia* ZOI” reflects the detection of inhibition of one or more *S. marcescens* target strains by secreted microcin in a zone of inhibition (ZOI) assay. For each microcin hit, the isolation source of the parent strain was determined. The percentage of hits per microcin from human vs nonhuman sources is displayed in a bar chart. A second bar chart displays percentages of different sources for the nonhuman fraction from the first bar chart. All residual microcins not attributed to one of the nine classes shown here are grouped into the “other” category. Significant enrichment of microcins in specific sources, based on chi-square tests, is indicated. Three microcin signal sequence variants not shown here were also significantly enriched: difsigSM017 (nonhuman samples; *P* < 0.001), difsigSM020 (human samples; *P* < 0.05), and difsig2SM064 (human samples; *P* < 0.05) (File S1). (**B**) Sources for putative class II microcins encoded by *S. marcescens* strains isolated from humans. For the top 10 most abundant microcins overall, the percentage of hits attributable to the top 5 human isolation sources (blood, respiratory, gastrointestinal, urinary, and wound) is indicated. Percentages >15% are noted per column. Significant enrichment of microcins in specific sources, based on chi-square tests, is indicated.

To illustrate the sequence relationships between the 40 cloned putative class II microcins and contextualize them in terms of the *in vitro* antibacterial activity analyses, we pruned the previous 93-microcin phylogeny (Fig. S1) to show only these 40 sequences and updated the numbers of hits per sequence identified using the improved microcin detection pipeline (9) (Fig. 3A). These 40 microcins accounted for 4,140 (86%) of the hits to the 93 unique microcin sequences. Summary columns adjacent to the phylogeny describe (i) inhibition of *E. coli* DH5α growth during secretion (+/-) and (ii) inhibition of any *S. marcescens* strain by ZOI assay (+/-) (Fig. 3A).

Interestingly, only two microcins (SM064 and SM079) both inhibited the growth of *E. coli* and produced a ZOI on *S. marcescens* (Fig. 3A). Both microcins belong to the same phylogenetic clade (clade X), indicating they have sequence similarity. Other phylogenetic patterns of antibacterial activity can be observed. For example, all tested microcins in clade IX (*n* = 3) inhibit the growth of *E. coli*, but no ZOI were observed against *S. marcescens*. There are also other cases where microcin pairs with sequence similarity are both active. For some clades (II, IV, VII), there was no observation of antibacterial activity for any microcin.

Due to the known diversity of sources and lifestyles of *S. marcescens*, we wanted to determine if there was a relationship between carriage of particular *S. marcescens* microcins and the isolation source of their strain of origin. We hypothesized that *S. marcescens* microcin type and prevalence vary by isolation source. To assess this, available metadata was used to assign a source category per strain: human, soil, hospital, wastewater, insect, coral, plant, mammal, water, mollusc, or other. The latter 10 categories are collectively referred to as “nonhuman” sources. Strains in the human category were further subcategorized: blood, respiratory, gastrointestinal, urinary, or wound. Accordingly, hits to all 93 putative class II microcins were assigned to their strain source (File S1).

For the 40 microcins tested *in vitro*, the percentage from human vs nonhuman *S. marcescens* isolates was plotted in a bar chart corresponding to microcin phylogenetics (Fig. 3A). A second bar chart subdivides the nonhuman group into its specific source category percentages per microcin. Most microcins (*n* = 3,886; 80.8%) were encoded in isolates from humans, reflecting the frequency of clinical strain isolation. However, distinct differences in carriage rate by isolation source are seen for individual microcins. Among the 14 most abundant microcins, 11 were significantly more abundant in either human (*n* = 5) or nonhuman (*n* = 6) isolates (File S1; Fig. 3A). The four most abundant microcins (SM027, SM051, SM017, and SM064) are more often found in human isolates (Fig. 3A) and are the most strongly co-associated (Fig. 1B). In contrast, other abundant microcins (SM003, SM002, SM052, SM040; >1% of microcin hits each) were significantly more frequent in nonhuman isolates (Fig. 3A). Microcin co-association data (Fig. 1B) correlates with these observations as well; SM003/SM052 and SM002/SM040 are co-associated microcin pairs. Some of the 40 microcins were only found in human isolates (*n* = 14), while some were never found in human isolates (*n* = 7). However, with four human-only exceptions (SM021, SM022, SM049, and SM042), these are low abundance microcins (<10 hits).

Among nonhuman-origin microcins, the top sources of origin for their parent strains were, in order, soil, hospital, wastewater, coral, plant, insect, mammal, mollusc, and water. Hospital and wastewater sources likely reflect human-associated strains to varying degrees; microcins with hospital and wastewater sources were also frequently found to originate from human samples. Microcins encoded by isolates from soil, plants, and insects are relatively dispersed throughout the phylogeny (Fig. 3A). *S. marcescens* strains from corals and molluscs contain a narrower selection of microcins (predominantly SM040 and SM079). There may be a geographic element to this observation, as all coral and mollusc isolates were collected in Florida, United States.

For the 3,037 human-origin microcins for which an isolation subcategory could be determined, the majority were attributed to blood (46%), respiratory (27%), gastrointestinal (5.9%), urinary (5.1%), or wound (5.1%) sources. For the 10 most abundant microcins overall, a bar chart shows the percentages of each per human source subcategory (Fig. 3B). Microcin abundance varied per isolation source, and three microcins (SM003, difsigSM017, and SM064) were significantly more abundant in specific human sources (Fig. 3B). SM064, which is significantly more abundant in human isolates (Fig. 3A), is significantly more abundant in blood samples (Fig. 3B). SM013, found predominantly in human-origin isolates (Fig. 3A), was the only microcin shown here that was never detected in all five subcategories, as no SM013 were encoded in gastrointestinal isolates (Fig. 3B).

### Relationships between *S. marcescens* target strain genomics and microcin sensitivity

**Fig 4:**
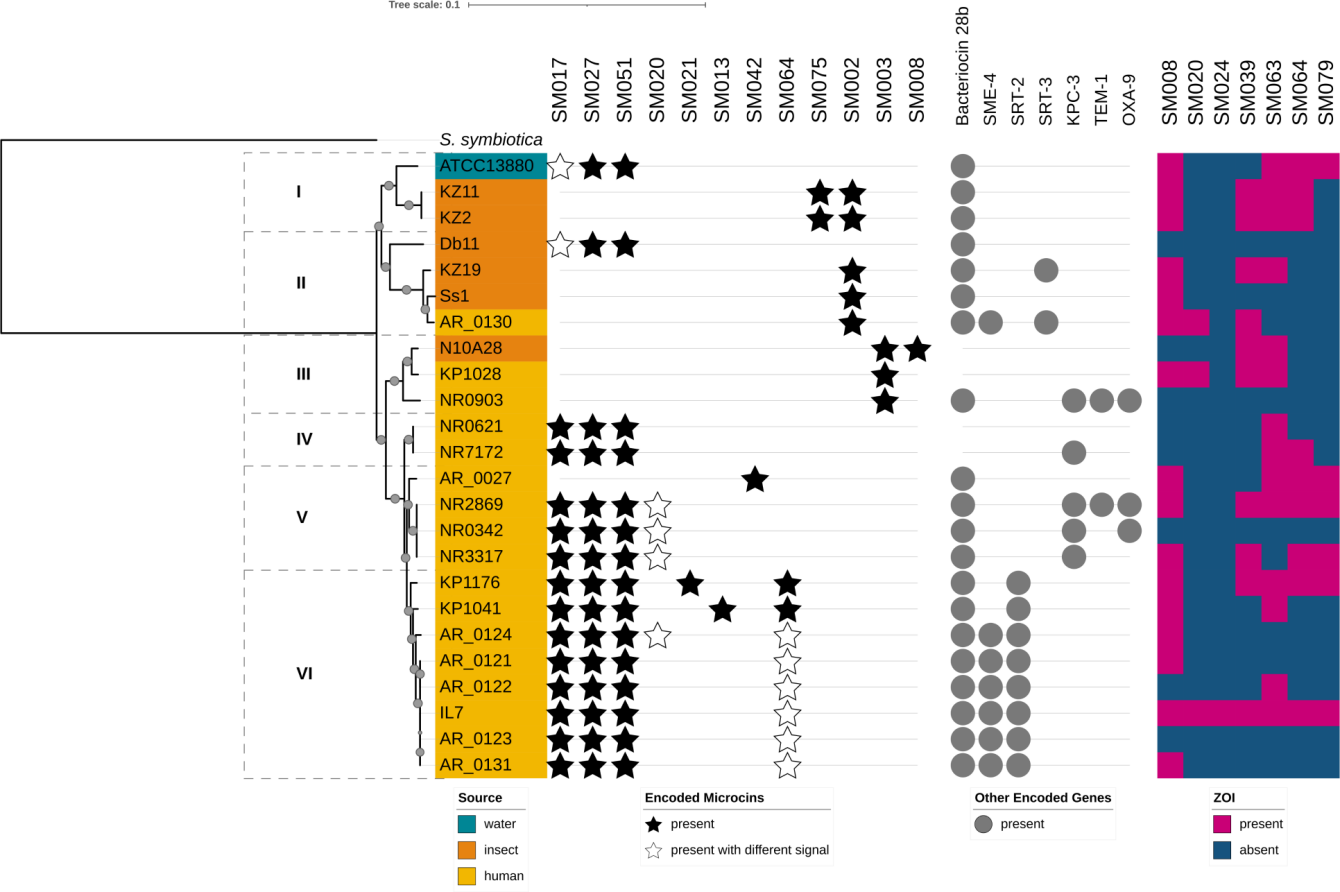
*Serratia marcescens* isolate phylogenomics vs class II microcin susceptibility. A panel of sequenced *S. marcescens* isolates (*n* = 24) was tested for susceptibility to a panel of *S. marcescens*-origin microcins (*n* = 7). A core genome phylogeny of the *S. marcescens* isolates was generated, with *Serratia symbiotica* as the outgroup. Bootstrap support values are 100% except for four terminal values in clades V and VI (range 61%–98%), indicated on the branches by their relatively smaller-sized gray circles. Major clades are indicated by roman numerals. Genomes were screened *in silico* for class II microcins, and microcin presence is indicated by a black star (encoded microcin) or white star (same encoded core microcin with different signal sequence). Genomes were also screened for the presence of another, larger *Serratia* bacteriocin (28b) and beta-lactamases (notably, SME-4 and KPC-3 are carbapenemases), as indicated by gray circles. Susceptibility of *S. marcescens* strains to class II microcins was determined via zone of inhibition (ZOI) assays, with microcins secreted heterologously from *E. coli*. Microcin susceptibility per strain is displayed in a heat map (pink = ZOI present, blue = ZOI absent).

Having looked at the phylogenetic relationships between *S. marcescens* class II microcins, we turned to the phylogenomic relationships between the 24 *S. marcescens* target strains analyzed here to understand their observed microcin susceptibility. Their genome assemblies were used to build a phylogeny, and major strain clades (I–VI) were annotated (Fig. 4). Adjacent to this phylogeny, the presence/absence of select genetic attributes and a heatmap of microcin sensitivity from ZOI assays are shown per strain (Fig. 4). *S. marcescens* phylogenomics appears to correspond to strain isolation source (Fig. 4), as shown previously with larger datasets (29, 30). Clades I–II contain insect/water isolates with a single exception, while Clades III–VI contain human clinical isolates with a single exception. There is not an obvious relationship between phylogenomics and microcin susceptibility by ZOI (Fig. 4). For example, the four isolates against which no microcin was active span the phylogeny. However, a larger isolate collection from more diverse sources may be needed to observe genomic patterns in microcin susceptibility.

Next, we examined the presence of natively encoded microcins in *S. marcescens* target strain genomes. These isolates encoded from 1 to 5 microcins (Fig. 4). Groups of closely related strains often carry the same microcins; e.g., all eight clade VI strains encode SM017, SM027, and SM051, which we have shown to be co-associated (Fig. 1B). Variations in microcin carriage are nonetheless observed among closely related strains. Using the same example of clade VI, only 5/8 strains encode an identical set of microcins. Collectively, these data suggest some relationship between isolate phylogenomics and carriage of particular microcin(s), though the relationship is not strict.

Because class II microcins are encoded adjacent to their cognate immunity protein to protect the producing strain, the presence of a specific microcin in a bacterial strain implies resistance to that microcin. Among the 24 *S. marcescens* target strains tested here, 12 strains collectively encoded 13 instances of 3 microcins (SM008, SM020, SM064) which produced a ZOI on one or more strains. Consistent with expectations, most of the ZOI-producing microcins did not produce a ZOI on the target strains in which they were encoded. However, for SM064, weak ZOI were observed on strains IL7 and KP1176 (Fig. 2B). The high level of induced SM064 secretion from the heterologous *E. coli* mT1SS likely overwhelmed the native immunity protein defenses.

Next, we inspected other antibacterial characteristics of the 24 *S. marcescens* strains. The only characterized bacteriocin produced by *S. marcescens* is bacteriocin 28b, a larger (47.5 kDa) colicin-like bacteriocin which is active toward *E. coli* (31, 32). Bacteriocin 28b is encoded by most of these *S. marcescens* strains, except for those in clades III and IV, where it is encoded in only one strain, which has only one microcin (Fig. 4). Additionally, we assessed these genomes for the presence of beta-lactamases, including carbapenemases, which are clinically relevant in the treatment of *S. marcescens* infections (Fig. 4; File S3). Class II microcins identified here have antibacterial activity against some multidrug- and carbapenem-resistant strains (Fig. 2B and 4).

## DISCUSSION

Class II microcins are remarkably prevalent in *Serratia marcescens*. In *E. coli*, the species where class II microcins were originally discovered (33) and which has long been understood to be the primary producer of microcins (34), our previous analysis of 1,224 genome assemblies found that 23.5% contained class II microcins, and 19.4% of these contained more than one microcin (5). Here, for *S. marcescens*, these values increased to 96% and 74%, respectively. Our additional analyses in other bacterial species (5) support the concept that *S. marcescens* has an extraordinary rate of microcin carriage. Given the wide array of secreted factors produced by *Serratia* spp. (23), perhaps this should not be surprising. *Serratia* secretes numerous enzymes (extracellular nuclease [35], chitinases [36], phospholipase [37], and hemolysin [38]) and antibacterial toxins (type VI secretion system effectors [39–43], prodigiosin [44], and the aforementioned bacteriocin 28b [31]). Among *Serratia* spp., *S. marcescens* has by far the largest accessory genome of any species (29), suggesting it is particularly prone to the carriage of nonessential genes that provide an adaptive advantage. Here, we have added class II microcins to the extensive *S. marcescens* secretome.

Given that multiple class II microcins are detected in the majority of *S. marcescens* genome assemblies analyzed here, and that bacteriocin co-association is a documented phenomenon, it is unsurprising that certain *S. marcescens* class II microcins tend to be co-associated with each other. Specific to class II microcins, strong co-association has been shown between microcins H47 (MccH47) and M (MccM) (45–48), as well as microcin V (MccV) and the larger *E. coli* bacteriocin, colicin Ia (45, 49). In gram-positive lactic acid bacteria (LAB), which are known for the production of multiple bacteriocins, it has been suggested that the sharing of biosynthetic machinery among different bacteriocins may ease the cellular burden of bacteriocin production (50). MccH47 and MccM share the same mT1SS export machinery (15), and this could be the case with class II microcins from *S. marcescens* and other species as well. Interestingly, however, many of the *S. marcescens* assemblies analyzed here encode more than one PCAT needed for secretion. The specificity of multiple encoded secretion systems for multiple encoded microcins remains to be determined.

Here, we confirmed antibacterial activity for 17 class II microcins from *S. marcescens*, either through detection of growth inhibition of a heterologous *E. coli* secretor or ZOI on target *S. marcescens* strains. There was not much overlap between activity against *E. coli* vs *S. marcescens*. These two species are not in the same family of bacteria, which should decrease their likelihood of possessing sufficiently conserved protein targets required for microcin antibacterial activity. Future work to validate native microcin secretion from *S. marcescens* strains and determine the environmental cues regulating secretion would be valuable next steps to begin examining these microcins in an ecological context. For the putative microcins that did not produce a ZOI, many reasons exist to impede observation of a ZOI. These include, but are not limited to, an appropriate target strain/species was not part of our panel, limited agar solubility and/or diffusability of a given microcin (51), ideal media composition for activity of novel microcins is unknown (1), incorrect prediction or impeded activity of the cognate immunity protein, and/or requirement of additional modification proteins (e.g., for class IIb microcins (52) for optimal antibacterial activity. Lack of secretion is unlikely to contribute to lack of ZOI. Our microcin secretion system (8, 9, 22), as well as similar versions from other groups (53–57), has been characterized to secrete a wide variety of heterologous, small proteins, with size (must be microcin-sized or smaller) being the primary limitation to secretion.

Phylogenomics of the genus *Serratia* shows strong alignment with ecological niche (29, 30). Because of this, we anticipated some association between the specific class II microcin(s) encoded by an *S. marcescens* strain and the strain isolation source, and indeed, this does seem to be the case. In general, however, there are no clear phylogenomic or genetic patterns selected here that appear to predict which microcins may be active against a target strain. The outer membrane receptors or other target strain proteins involved in the mechanism of action of these *S. marcescens* microcins have not been identified; these as well as other nonspecific target strain factors including cell surface structures (e.g., capsule polysaccharides) could affect microcin susceptibility.

Certain class II microcins analyzed here are more abundant among nonhuman isolation sources than human isolation sources. However, microcin prevalence, sequence selection, and antibacterial activity characterization presented here is biased by heavy overrepresentation of human clinical isolates. Though *S. marcescens* is widely dispersed among different environments and hosts, the vast majority of genome-sequenced isolates are of clinical origin. The fact that the two broadest antibacterial range *S. marcescens* microcins targeting *S. marcescens* strains were infrequently detected in sequence data and, when they were, were mostly found in nonhuman source isolates, suggests there is value in mining for likely additional unexplored microcin diversity in *S. marcescens* from nonhuman sources.

## MATERIALS AND METHODS

### Putative class II microcin screening and selection *in silico*

*Serratia marcescens* (NCBI:txid615) GenBank genome assemblies (*n* = 1,656) were downloaded on 11/18/22 (File S1) and screened for class II microcins using our microcin identification pipeline, cinful v1 (5). Matches to the microcin pHMM were considered putative microcins. Review of assembly metadata downloaded from NCBI data sets identified 36 assemblies of *S. marcescens* type strain ATCC 13,880; 35 redundant assemblies (File S1) and their microcin hits were removed from downstream analyses. A multiple sequence alignment of all unique putative microcin amino acid sequences terminating in a stop codon (*n* = 93) and a gram-positive double-glycine bacteriocin outgroup (pediocin PA-1 and piscicolin-126) was generated with MAFFT v7.490 (58). Microcins containing excess predicted N-terminal sequence prior to the putative 15–18 amino acid double-glycine signal sequence (SM067-SM068, SM074-SM080, and difsigSM080) were trimmed to the presumptive start codon (File S1). A phylogeny (Fig. S1) was generated from the trimmed 95-taxon alignment with RAxML v8.2.11 (59) using the GAMMA WAG (60) protein model, rapid bootstrapping (*n* = 1,000), and a search for the best-scoring ML tree. Branches with low support values (<50%) were collapsed using Dendroscope 3.8.8 (61). The tree was visualized in Interactive Tree of Life (iTOL) v6.9.1 (62) and annotated using the iTOL annotation editor. Based on this phylogeny, 40 unique core microcin sequences were manually selected for cloning, including sequences that were highly abundant, evenly distributed across the phylogeny, and rare but phylogenetically distinct.

We subsequently developed an improved class II microcin detection pipeline, cinful v2 (9), and re-analyzed the same 1,621 *S. marcescens* assemblies for the presence of the 93 unique microcin sequences identified with cinful v1. Except for the initial analysis (Fig. S1), all described microcin data come from this re-analysis with cinful v2. Cinful v2 identified some additional putative microcins (File S1), but these were excluded from additional analysis here, which was focused on the original set of 93 hits. The 95-taxon phylogeny (Fig. S1) was pruned to show only the 40 putative microcin sequences selected for cloning and the outgroup (Fig. 3A). For *S. marcescens* assemblies containing one or more of the 93 unique microcins as determined using cinful v2 (9), *in silico* screening for the PCAT of the mT1SS was conducted using cinful v1 (5), which can differentiate PCATs from similar proteins lacking the necessary peptidase domain (63, 64).

### Assembly metadata curation

For all genome assemblies (*n* = 1,562) containing one or more of the 93 unique putative class II microcin sequences identified with cinful v2 (9), metadata from NCBI data sets and associated publications, if available, were manually reviewed and curated to classify the source of each *S. marcescens* strain (File S1). Primary source categories with >10 observed microcin hits are represented in downstream analyses. All other sources and unknown sources were grouped together as “other.” For the “human” primary category, secondary source categories with >100 observed microcins were also assigned (File S1).

Pearson’s chi-square tests were used to determine if putative microcins were encoded in *S. marcescens* strains from specific sources more often than expected by chance. Tests were conducted for human vs all other primary sources (nonhuman and other), which included the entire microcin data set (*n* = 4,809 hits), and for the five human secondary categories with >100 observed microcin hits per category (*n* = 2,703 hits). Standardized chi-squared residuals were converted to *P*-values, which were then adjusted for multiple comparisons using the Benjamini-Hochberg (BH) method for false discovery rate (FDR) correction. An encoded putative microcin and a strain isolation source were considered dependent if computed expected frequencies for all sources were ≥5 and the adjusted chi-squared *P* < 0.05.

### Class II microcin co-association

Multiple unique microcins per genome assembly were observed, so co-association between pairs of microcins was assessed. Hit counts per microcin from cinful v2 (9) were tabulated per assembly. In 51 instances, the same microcin sequence was detected in two different locations within a genome assembly. It is unknown if this is a true duplication event or an assembly artifact. For the purposes of counts of unique microcins per assembly and co-association analysis, microcin duplications were reduced to a single count per assembly. The number of unique microcin sequences observed per genome was summed. Contingency tables for pairs of microcins were generated, and co-association was assessed by chi-squared tests. Pearson’s chi-squared tests with Yates’ continuity correction, adjusted with the BH method for FDR correction, were computed for microcin presence/absence data per the 1,562 genome assemblies with microcins, for a total of 4,278 paired microcin combinations. Microcins were considered co-associated if the contingency table counts for all cells for the computed expected frequencies for a microcin pair were ≥5, the odds ratio computed from the observed frequencies was >1 (indicating a positive relationship), and the adjusted chi-squared *P* < 0.05. Co-associations were visualized in the R package igraph v 2.1.4 (65).

### Microcin antibacterial activity screening

The 40 putative microcins selected for antibacterial activity testing were cloned into arabinose-inducible pBAD18Km for secretion from our gram-negative bacterial microcin secretion system, as described (9, 22). The putative double-glycine signal sequence for each microcin (File S1) was replaced with the MccV signal sequence (cvaC15) used in the *E. coli* mT1SS (22). Gene synthesis and cloning were performed by Azenta Life Sciences. *E. coli* DH5α, a K-12 derivative cloning strain, was transformed with a pBAD18Km microcin construct and the constitutively expressed gram-negative microcin secretion system (pACYC184 CvaAB) (22). Growth curves to test for self-inhibition of the *E. coli* DH5α microcin secretor in the absence of the cognate immunity protein were performed in M9 minimal medium on a BioTek LogPhase 600 Microbiology Reader (Agilent) as described (9). OD600 was measured every 10 min for 6 h, with and without induction of microcin expression by 0.4% arabinose. Duplicate transformants were assayed in duplicate, for a total of four growth curves per treatment. Area under the curve (AUC) was computed per growth curve and compared between induced vs uninduced treatments for each microcin by Wilcoxon rank-sum tests, as we have done previously (9). If the mean AUC (induced) was less than the mean AUC (uninduced) and *P* < 0.05, the microcin was considered antibacterial toward *E. coli*. For microcins which inhibited the growth of *E. coli* when secreted (*n* = 12) and select additional microcins (*n* = 9), we followed our previous methods (9) to predict cognate immunity protein sequences (File S1) by manually searching for open reading frames encoded adjacent to each microcin and cloning them into the pBAD18Km constructs. Immunity proteins are named by adding “imm” to the name of the microcin. For two microcins (SM031 and SM033), two candidate immunity proteins were tested (e.g., immSM031 and imm2SM031).

Zone of inhibition (ZOI) assays were performed with *E. coli* DH5α microcin secretor strains individually spotted onto a lawn of an *S. marcescens* target strain of interest (Table S1). *E. coli* secretor strains expressing a microcin only (*n* = 40) or a microcin + cognate immunity protein (*n* = 23) were analyzed. ZOI assays were performed in M9 minimal media as described (9). Briefly, *E. coli* microcin secretors (+/- immunity) were concentrated to OD600 = 100, and 10 μL was spotted on a M9 soft agar overlay containing target cells at OD600 = 0.01% + 0.2% arabinose. Plates were incubated at 37°C overnight. ZOI assays were repeated at least in triplicate. For microcins where both +/- immunity secretor strains were available, the variant which produced qualitatively clearer, more easily detectable ZOI (Fig. S2), likely an indication of superior secretion, was used for final activity screening analyses.

### Split luciferase complementation assay

In an adaptation of previous work (28), the large fragment (11S) and small fragment (pep86) of split luciferase were cloned for expression from two separate vectors. For periplasmic export of 11S, we cloned pBAD malE-11S (Kan^R^), where 11S is fused to the sec secretion system signal of MalE (28). For extracellular secretion of pep86, we cloned pMMB67EH pep86-G3P2 + CvaAB (Amp^R^), for secretion from the one-plasmid version of the gram-negative secretion system (22). Here, pep86 is fused to the well-secreted nonsense peptide, G3P2, to increase the total cargo size to one appropriate for the secretion system (22). Both plasmids were transformed separately or together into *E. coli* DH5α.

Overnight cultures of *E. coli* DH5α strains (100 µL) were added to fresh LB media plus antibiotics, and cultures were grown at 37°C until early exponential growth phase. Cultures were treated for 2 h with 0.2% arabinose to induce the expression of 11S. Cultures were centrifuged (4,000 rpm, 10 min), supernatant was removed, and pellets were resuspended in LB medium without antibiotics (OD600 = 0.5). The expression of pep86 was induced at three different IPTG concentrations (2 µM, 20 µM, or 200 µM) for 20 min. A bioluminescent reporter assay, Nano-Glo Live Cell Assay System (Promega), was used to measure luciferase activity in a 96-well Black Polystyrene Non-Binding Flat Bottom Microplate (Greiner Bio-One); resulting luminescence (relative light units, RLU) was detected using the BioTek Synergy LX Multimode Reader (Agilent). One-way ANOVA with *post hoc* multiple comparisons was conducted to compare treatments.

### Target strain genome analyses

Genome assemblies from the *S. marcescens* isolates used for microcin *in vitro* activity screening (*n* = 24, Table S1) were previously available except for *S. marcescens* IL7, KP1028, KP1041, KP1176, NR0342, NR0621, NR0903, NR2869, NR3317, and NR7172. DNA was isolated from these isolates using the DNeasy Blood & Tissue Kit (QIAGEN) following the protocol for gram-negative bacteria. Whole bacterial genome sequencing of *S. marcescens* IL7 was performed by Plasmidsaurus using Oxford Nanopore Technologies (ONT). For the other 9 isolates, a 1.5× bead-cleanup was performed after genomic DNA extraction to purify the extraction and remove any small fragments that would not be compatible with long-read sequencing. Unique index-tagged libraries consisting of 600 bp paired-end reads were generated for each isolate following the Illumina DNA library preparation kit (Illumina) for short-read WGS. Additionally, the same genomic DNA was prepared using the Rapid Barcoding Sequencing Kit (SQK-RBK004) for long-read WGS (ONT). Sequencing was performed using an Illumina MiSeq instrument and an ONT GridION using R9 flow cells, respectively, at the Columbia Microbiome and Pathogen Genomics Core. For ONT, basecalling and demultiplexing were performed using the MinKNOW program (ONT). Hybrid *de novo* assembly performed using Unicycler v0.4.8 (66) was followed by annotation using Prokka v1.12 (67) and BLAST manual curation as previously described (68). Target strain genomes were screened for the 93 unique putative class II microcins terminating in a stop codon identified in our original *in silico* screen of genome assemblies using cinful (5). A tblastn search of the 93 putative microcin amino acid sequences against the 24 genome assemblies was performed using default parameters, and identical hits were retained and summarized using the same microcin IDs described during the initial genome screen (File S1). Target strain genomes were screened for antibiotic resistance determinants using the ARG-ANNOT database (69) and the Comprehensive Antibiotic Resistance Database (CARD) (70) via SRST2 (71). Only “Perfect” hits identified by CARD are reported in the figures. Complete CARD outputs for “Perfect” and “Strict” hits are available in File S3.

A phylogeny was generated from the 24 genome assemblies following the published *S. marcescens* cgMLST scheme (72), with *Serratia symbiotica* CWBI-2.3 (GCA_000821185.2) as the outgroup genome (12). In the cgMLST scheme, allele 1 for each of the 2,692 included genes is from *S. marcescens* Db11 (72). Per gene, megablast of allele 1 was used to retrieve the gene homologs (max *E*-value 0.05) in the 25 *Serratia* spp. genomes. For all genes where mean coverage = 25 for the alignment and a single hit per genome was retrieved (*n* = 492), the gene alignments were concatenated. The concatenated alignment (498,847 bp) was used to generate a phylogeny using RAxML (59) with the GTR CAT nucleotide model, rapid bootstrapping (*n* = 1,000), and a search for best-scoring ML tree. Branches with <50% support were collapsed using Dendroscope and the tree (Fig. 4) visualized and annotated in iTOL as described above.

## Data Availability

Genome assemblies are available at NCBI under the following accessions: CP170117 and SAMN52858952-SAMN52858960 (Table S1). Microcin sequences (File S1), microcin activity data (File S2), and CARD output (File S3) are available as supplemental material.

## SUPPLEMENTAL MATERIAL

File S1 aem.00259-26-s0001.xlsx Microcin sequences.

File S2 aem.00259-26-s0002.xlsx Microcin activity data.

File S3 aem.00259-26-s0003.xlsx CARD output.

Supplemental material aem.00259-26-s0004.docx Fig. S1 to S3 and Table S1.

ASM does not own the copyrights to Supplemental Material that may be linked to, or accessed through, an article. The authors have granted ASM a non-exclusive, world-wide license to publish the Supplemental Material files. Please contact the corresponding author directly for reuse.

## References

[1] Sassone-Corsi M, Nuccio S-P, Liu H, Hernandez D, Vu CT, Takahashi AA, Edwards RA, Raffatellu M. 2016. Microcins mediate competition among Enterobacteriaceae in the inflamed gut. Nature 540:280–283. doi:10.1038/nature20557 27798599 PMC5145735

[2] Mortzfeld BM, Palmer JD, Bhattarai SK, Dupre HL, Mercado-Lubio R, Silby MW, Bang C, McCormick BA, Bucci V. 2022. Microcin MccI47 selectively inhibits enteric bacteria and reduces carbapenem-resistant. Gut Microbes 14. doi:10.1080/19490976.2022.2127633 PMC9542533 36175830

[3] Wooley RE, Gibbs PS, Shotts EB. 1999. Inhibition of Salmonella typhimurium in the chicken intestinal tract by a transformed avirulent avian Escherichia coli. Avian Dis 43:245–250. doi:10.2307/1592614 10396637

[4] Parker JK, Davies BW. 2022. Microcins reveal natural mechanisms of bacterial manipulation to inform therapeutic development. Microbiology (Reading) 168:001175. doi:10.1099/mic.0.001175 35438625 PMC10233263

[5] Cole TJ, Parker JK, Feller AL, Wilke CO, Davies BW. 2022. Evidence for widespread class II microcins in Enterobacterales genomes. Appl Environ Microbiol 88:e0148622. doi:10.1128/aem.01486-22 36394322 PMC9746304

[6] Kulikova AV, Parker JK, Davies BW, Wilke CO. 2024. Semantic search using protein large language models detects class II microcins in bacterial genomes. mSystems 9:e0104424. doi:10.1128/msystems.01044-24 39291976 PMC11494933

[7] Bisaro F, Shuman HA, Feldman MF, Gebhardt MJ, Pukatzki S. 2023. Acinetobacter baumannii ATCC 17978 encodes a microcin system with antimicrobial properties for contact-independent competition. Microbiology (Reading) 169:001346. doi:10.1099/mic.0.001346 37289493 PMC10333792

[8] Kim SY, Randall JR, Gu R, Nguyen QD, Davies BW. 2024. Antibacterial action, proteolytic immunity, and in vivo activity of a Vibrio cholerae microcin. Cell Host Microbe 32:1959–1971. doi:10.1016/j.chom.2024.08.012 39260372 PMC11563924

[9] Parker JK, Feller AL, Gu R, Sanchez-Paiva S, Perez BC, O’Donnell AC, Deng W, Ousterhout RM, Kim S-Y, Wilke CO, Davies BW. 2025. Antibacterial microcins are ubiquitous and functionally diverse across bacterial communities. Nat Commun 16:6048. doi:10.1038/s41467-025-61151-z 40595659 PMC12219008

[10] Iguchi A, Nagaya Y, Pradel E, Ooka T, Ogura Y, Katsura K, Kurokawa K, Oshima K, Hattori M, Parkhill J, Sebaihia M, Coulthurst SJ, Gotoh N, Thomson NR, Ewbank JJ, Hayashi T. 2014. Genome evolution and plasticity of Serratia marcescens, an important multidrug-resistant nosocomial pathogen. Genome Biol Evol 6:2096–2110. doi:10.1093/gbe/evu160 25070509 PMC4231636

[11] Mahlen SD. 2011. Serratia infections: from military experiments to current practice. Clin Microbiol Rev 24:755–791. doi:10.1128/CMR.00017-11 21976608 PMC3194826

[12] Raymann K, Coon KL, Shaffer Z, Salisbury S, Moran NA. 2018. Pathogenicity of Serratia marcescens strains in honey bees. mBio 9:e01649-18. doi:10.1128/mBio.01649-18 30301854 PMC6178626

[13] Sutherland KP, Shaban S, Joyner JL, Porter JW, Lipp EK. 2011. Human pathogen shown to cause disease in the threatened eklhorn coral Acropora palmata. PLoS One 6:e23468. doi:10.1371/journal.pone.0023468 21858132 PMC3157384

[14] Duquesne S, Destoumieux-Garzón D, Peduzzi J, Rebuffat S. 2007. Microcins, gene-encoded antibacterial peptides from enterobacteria. Nat Prod Rep 24:708–734. doi:10.1039/b516237h 17653356

[15] Vassiliadis G, Destoumieux-Garzón D, Lombard C, Rebuffat S, Peduzzi J. 2010. Isolation and characterization of two members of the siderophore-microcin family, microcins M and H47. Antimicrob Agents Chemother 54:288–297. doi:10.1128/AAC.00744-09 19884380 PMC2798501

[16] Hodges FJ, Torres VVL, Cunningham AF, Henderson IR, Icke C. 2023. Redefining the bacterial Type I protein secretion system. Adv Microb Physiol 82:155–204. doi:10.1016/bs.ampbs.2022.10.003 36948654

[17] Håvarstein LS, Diep DB, Nes IF. 1995. A family of bacteriocin ABC transporters carry out proteolytic processing of their substrates concomitant with export. Mol Microbiol 16:229–240. doi:10.1111/j.1365-2958.1995.tb02295.x 7565085

[18] Gilson L, Mahanty HK, Kolter R. 1990. Genetic analysis of an MDR-like export system: the secretion of colicin V. EMBO J 9:3875–3884. doi:10.1002/j.1460-2075.1990.tb07606.x 2249654 PMC552155

[19] Azpiroz MF, Rodríguez E, Laviña M. 2001. The structure, function, and origin of the microcin H47 ATP-binding cassette exporter indicate its relatedness to that of colicin V. Antimicrob Agents Chemother 45:969–972. doi:10.1128/AAC.45.3.969-972.2001 11181394 PMC90407

[20] Hwang J, Zhong X, Tai PC. 1997. Interactions of dedicated export membrane proteins of the colicin V secretion system: CvaA, a member of the membrane fusion protein family, interacts with CvaB and TolC. J Bacteriol 179:6264–6270. doi:10.1128/jb.179.20.6264-6270.1997 9335271 PMC179538

[21] Azpiroz MF, Laviña M. 2004. Involvement of enterobactin synthesis pathway in production of microcin H47. Antimicrob Agents Chemother 48:1235–1241. doi:10.1128/AAC.48.4.1235-1241.2004 15047525 PMC375329

[22] Kim SY, Parker JK, Gonzalez-Magaldi M, Telford MS, Leahy DJ, Davies BW. 2023. Export of diverse and bioactive small proteins through a type I secretion system. Appl Environ Microbiol 89:e0033523. doi:10.1128/aem.00335-23 37078870 PMC10231218

[23] Petersen LM, Tisa LS. 2013. Friend or foe? A review of the mechanisms that drive Serratia towards diverse lifestyles. Can J Microbiol 59:627–640. doi:10.1139/cjm-2013-0343 24011346

[24] Adeolu M, Alnajar S, Naushad S, S Gupta R. 2016. Genome-based phylogeny and taxonomy of the ‘Enterobacteriales’: proposal for Enterobacterales ord. nov. divided into the families Enterobacteriaceae, Erwiniaceae fam. nov., Pectobacteriaceae fam. nov., Yersiniaceae fam. nov., Hafniaceae fam. nov., Morganellaceae fam. nov., and Budviciaceae fam. nov. Int J Syst Evol Microbiol 66:5575–5599. doi:10.1099/ijsem.0.001485 27620848

[25] Monk JM, Koza A, Campodonico MA, Machado D, Seoane JM, Palsson BO, Herrgård MJ, Feist AM. 2016. Multi-omics quantification of species variation of Escherichia coli links molecular features with strain phenotypes. Cell Syst 3:238–251. doi:10.1016/j.cels.2016.08.013 27667363 PMC5058344

[26] Azpiroz MF, Laviña M. 2007. Modular structure of microcin H47 and colicin V. Antimicrob Agents Chemother 51:2412–2419. doi:10.1128/AAC.01606-06 17452478 PMC1913283

[27] Zhang LH, Fath MJ, Mahanty HK, Tai PC, Kolter R. 1995. Genetic analysis of the colicin V secretion pathway. Genetics 141:25–32. doi:10.1093/genetics/141.1.25 8536973 PMC1206723

[28] Wagstaff JM, Balmforth M, Lewis N, Dods R, Rowland C, van Rietschoten K, Chen L, Harrison H, Skynner MJ, Dawson M, Ivanova-Berndt G, Beswick P. 2020. An assay for periplasm entry advances the development of chimeric peptide antibiotics. ACS Infect Dis 6:2355–2361. doi:10.1021/acsinfecdis.0c00389 32697574

[29] Williams DJ, Grimont PAD, Cazares A, Grimont F, Ageron E, Pettigrew KA, Cazares D, Njamkepo E, Weill F-X, Heinz E, Holden MTG, Thomson NR, Coulthurst SJ. 2022. The genus Serratia revisited by genomics. Nat Commun 13:5195. doi:10.1038/s41467-022-32929-2 36057639 PMC9440931

[30] Shikov AE, Merkushova AV, Savina IA, Nizhnikov AA, Antonets KS. 2023. The man, the plant, and the insect: shooting host specificity determinants in Serratia marcescens pangenome. Front Microbiol 14:1211999. doi:10.3389/fmicb.2023.1211999 38029097 PMC10656689

[31] Viejo MB, Gargallo D, Ferrer S, Enfedaque J, Regué M. 1992. Cloning and DNA sequence analysis of a bacteriocin gene of Serratia marcescens. J Gen Microbiol 138 Pt 8:1737–1743. doi:10.1099/00221287-138-8-1737 1527512

[32] Guasch JF, Enfedaque J, Ferrer S, Gargallo D, Regué M. 1995. Bacteriocin 28b, a chromosomally encoded bacteriocin produced by most Serratia marcescens biotypes. Res Microbiol 146:477–483. doi:10.1016/0923-2508(96)80293-2 8525064

[33] Gratia A. 1925. Sur un remarquable exemple d’antagonisme entre deux souches de colibacille. Compt Rend Soc Biol 93:1040–1041.

[34] Baquero F, Lanza VF, Baquero MR, Del Campo R, Bravo-Vázquez DA. 2019. Microcins in Enterobacteriaceae: peptide antimicrobials in the eco-active intestinal chemosphere. Front Microbiol 10:2261. doi:10.3389/fmicb.2019.02261 31649628 PMC6795089

[35] Benedik MJ, Strych U. 1998. Serratia marcescens and its extracellular nuclease. FEMS Microbiol Lett 165:1–13. doi:10.1111/j.1574-6968.1998.tb13120.x 9711834

[36] Costa M de A, Owen RA, Tammsalu T, Buchanan G, Palmer T, Sargent F. 2019. Controlling and co-ordinating chitinase secretion in a Serratia marcescens population. Microbiology (Reading, Engl) 165:1233–1244. doi:10.1099/mic.0.000856 31526448

[37] Anderson MT, Mitchell LA, Mobley HLT. 2017. Cysteine biosynthesis controls Serratia marcescens phospholipase activity. J Bacteriol 199:e00159-17. doi:10.1128/JB.00159-17 28559296 PMC5527384

[38] Braun V, Ondraczek R, Hobbie S. 1993. Activation and secretion of Serratia hemolysin. Zentralbl Bakteriol 278:306–315. doi:10.1016/s0934-8840(11)80847-9 8347934

[39] Murdoch SL, Trunk K, English G, Fritsch MJ, Pourkarimi E, Coulthurst SJ. 2011. The opportunistic pathogen Serratia marcescens utilizes type VI secretion to target bacterial competitors. J Bacteriol 193:6057–6069. doi:10.1128/JB.05671-11 21890705 PMC3194891

[40] Fritsch MJ, Trunk K, Diniz JA, Guo M, Trost M, Coulthurst SJ. 2013. Proteomic identification of novel secreted antibacterial toxins of the Serratia marcescens type VI secretion system. Mol Cell Proteomics 12:2735–2749. doi:10.1074/mcp.M113.030502 23842002 PMC3790287

[41] Mariano G, Trunk K, Williams DJ, Monlezun L, Strahl H, Pitt SJ, Coulthurst SJ. 2019. A family of Type VI secretion system effector proteins that form ion-selective pores. Nat Commun 10:5484. doi:10.1038/s41467-019-13439-0 31792213 PMC6889166

[42] Jiang L, Yi W, Zhao Y, Zhu N, Zhao D, Peng Z, Song L, Dong T, Jiang X, Liu D, Ji X, Guan Q, Jiang H. 2025. Comprehensive genomic analysis of type VI secretion system diversity and associated proteins in Serratia. Microb Genom 11:001424. doi:10.1099/mgen.0.001424 40512661 PMC12165300

[43] Alcoforado Diniz J, Coulthurst SJ. 2015. Intraspecies competition in Serratia marcescens is mediated by type VI-secreted Rhs effectors and a conserved effector-associated accessory protein. J Bacteriol 197:2350–2360. doi:10.1128/JB.00199-15 25939831 PMC4524185

[44] Yip CH, Mahalingam S, Wan KL, Nathan S. 2021. Prodigiosin inhibits bacterial growth and virulence factors as a potential physiological response to interspecies competition. PLoS One 16:e0253445. doi:10.1371/journal.pone.0253445 34161391 PMC8221495

[45] Gordon DM, O’Brien CL. 2006. Bacteriocin diversity and the frequency of multiple bacteriocin production in Escherichia coli. Microbiology (Reading) 152:3239–3244. doi:10.1099/mic.0.28690-0 17074895

[46] Azpiroz MF, Poey ME, Laviña M. 2009. Microcins and urovirulence in Escherichia coli. Microb Pathog 47:274–280. doi:10.1016/j.micpath.2009.09.003 19744552

[47] Abraham S, Chapman TA, Zhang R, Chin J, Mabbett AN, Totsika M, Schembri MA. 2012. Molecular characterization of Escherichia coli strains that cause symptomatic and asymptomatic urinary tract infections. J Clin Microbiol 50:1027–1030. doi:10.1128/JCM.06671-11 22189125 PMC3295173

[48] Massip C, Chagneau CV, Boury M, Oswald E. 2020. The synergistic triad between microcin, colibactin, and salmochelin gene clusters in uropathogenic Escherichia coli. Microbes Infect 22:144–147. doi:10.1016/j.micinf.2020.01.001 31954842

[49] Jeziorowski A, Gordon DM. 2007. Evolution of microcin V and colicin Ia plasmids in Escherichia coli. J Bacteriol 189:7045–7052. doi:10.1128/JB.00243-07 17644607 PMC2045219

[50] Perez RH, Zendo T, Sonomoto K. 2022. Multiple bacteriocin production in lactic acid bacteria. J Biosci Bioeng 134:277–287. doi:10.1016/j.jbiosc.2022.07.007 35927130

[51] Bauer AW, Kirby WM, Sherris JC, Turck M. 1966. Antibiotic susceptibility testing by a standardized single disk method. Tech Bull Regist Med Technol 36:49–52. doi:10.1093/ajcp/45.4_ts.493 5908210

[52] Thomas X, Destoumieux-Garzón D, Peduzzi J, Afonso C, Blond A, Birlirakis N, Goulard C, Dubost L, Thai R, Tabet JC, Rebuffat S. 2004. Siderophore peptide, a new type of post-translationally modified antibacterial peptide with potent activity. J Biol Chem 279:28233–28242. doi:10.1074/jbc.M400228200 15102848

[53] Flórez V, Marizcurrena J, Laviña M, Azpiroz MF. 2024. Secretion of the human parathyroid hormone through a microcin type I secretion system in Escherichia coli. Microb Cell Fact 23:273. doi:10.1186/s12934-024-02552-5 39390566 PMC11465617

[54] Geldart K, Forkus B, McChesney E, McCue M, Kaznessis YN. 2016. pMPES: a modular peptide expression system for the delivery of antimicrobial peptides to the site of gastrointestinal infections using probiotics. Pharmaceuticals (Basel) 9:60. doi:10.3390/ph9040060 27782051 PMC5198035

[55] Kim H, Jang JH, Jung IY, Kim HR, Cho JH. 2023. Novel genetically engineered probiotics for targeted elimination of Pseudomonas aeruginosa in intestinal colonization. Biomedicines 11:2645. doi:10.3390/biomedicines11102645 37893018 PMC10604247

[56] van Belkum MJ, Worobo RW, Stiles ME. 1997. Double-glycine-type leader peptides direct secretion of bacteriocins by ABC transporters: colicin V secretion in Lactococcus lactis. Mol Microbiol 23:1293–1301. doi:10.1046/j.1365-2958.1997.3111677.x 9106219

[57] Geldart KG, Kommineni S, Forbes M, Hayward M, Dunny GM, Salzman NH, Kaznessis YN. 2018. Engineered E. coli Nissle 1917 for the reduction of vancomycin-resistant Enterococcus in the intestinal tract. Bioeng Transl Med 3:197–208. doi:10.1002/btm2.10107 30377660 PMC6195901

[58] Katoh K, Standley DM. 2013. MAFFT multiple sequence alignment software version 7: improvements in performance and usability. Mol Biol Evol 30:772–780. doi:10.1093/molbev/mst010 23329690 PMC3603318

[59] Stamatakis A. 2014. RAxML version 8: a tool for phylogenetic analysis and post-analysis of large phylogenies. Bioinformatics 30:1312–1313. doi:10.1093/bioinformatics/btu033 24451623 PMC3998144

[60] Whelan S, Goldman N. 2001. A general empirical model of protein evolution derived from multiple protein families using a maximum-likelihood approach. Mol Biol Evol 18:691–699. doi:10.1093/oxfordjournals.molbev.a003851 11319253

[61] Huson DH, Richter DC, Rausch C, Dezulian T, Franz M, Rupp R. 2007. Dendroscope: an interactive viewer for large phylogenetic trees. BMC Bioinformatics 8:460. doi:10.1186/1471-2105-8-460 18034891 PMC2216043

[62] Letunic I, Bork P. 2021. Interactive Tree Of Life (iTOL) v5: an online tool for phylogenetic tree display and annotation. Nucleic Acids Res 49:W293–W296. doi:10.1093/nar/gkab301 33885785 PMC8265157

[63] Wu KH, Tai PC. 2004. Cys^32^ and His^105^ are the critical residues for the calcium-dependent cysteine proteolytic activity of CvaB, an ATP-binding cassette transporter. J Biol Chem 279:901–909. doi:10.1074/jbc.M308296200 14570918

[64] Wu KH, Hsieh YH, Tai PC. 2012. Mutational analysis of Cvab, an ABC transporter involved in the secretion of active colicin V. PLoS One 7:e35382. doi:10.1371/journal.pone.0035382 22539970 PMC3335142

[65] Csardi G, Nepusz T. 2006. The igraph software package for complex network research. InterJournal Complex Systems. https://igraph.org.

[66] Wick RR, Judd LM, Gorrie CL, Holt KE. 2017. Unicycler: resolving bacterial genome assemblies from short and long sequencing reads. PLoS Comput Biol 13:e1005595. doi:10.1371/journal.pcbi.1005595 28594827 PMC5481147

[67] Seemann T. 2014. Prokka: rapid prokaryotic genome annotation. Bioinformatics 30:2068–2069. doi:10.1093/bioinformatics/btu153 24642063

[68] Giddins MJ, Macesic N, Annavajhala MK, Stump S, Khan S, McConville TH, Mehta M, Gomez-Simmonds A, Uhlemann AC. 2018. Successive emergence of ceftazidime-avibactam resistance through distinct genomic adaptations in bla_KPC-2_-harboring Klebsiella pneumoniae sequence type 307 isolates. Antimicrob Agents Chemother 62:e02101-17. doi:10.1128/AAC.02101-17 29263067 PMC5826117

[69] Gupta SK, Padmanabhan BR, Diene SM, Lopez-Rojas R, Kempf M, Landraud L, Rolain JM. 2014. ARG-ANNOT, a new bioinformatic tool to discover antibiotic resistance genes in bacterial genomes. Antimicrob Agents Chemother 58:212–220. doi:10.1128/AAC.01310-13 24145532 PMC3910750

[70] Alcock BP, Huynh W, Chalil R, Smith KW, Raphenya AR, Wlodarski MA, Edalatmand A, Petkau A, Syed SA, Tsang KK, et al.. 2023. CARD 2023: expanded curation, support for machine learning, and resistome prediction at the Comprehensive Antibiotic Resistance Database. Nucleic Acids Res 51:D690–D699. doi:10.1093/nar/gkac920 36263822 PMC9825576

[71] Inouye M, Dashnow H, Raven LA, Schultz MB, Pope BJ, Tomita T, Zobel J, Holt KE. 2014. SRST2: rapid genomic surveillance for public health and hospital microbiology labs. Genome Med 6:90. doi:10.1186/s13073-014-0090-6 25422674 PMC4237778

[72] Kampmeier S, Prior K, Cunningham SA, Goyal A, Harmsen D, Patel R, Mellmann A. 2022. Development and evaluation of a core genome multilocus sequencing typing (cgMLST) scheme for Serratia marcescens molecular surveillance and outbreak investigations. J Clin Microbiol 60:e0119622. doi:10.1128/jcm.01196-22 36214584 PMC9667775

