# Supplementary material for: Prevalence and activity of class II microcins in Serratia marcescens strains by isolation source
Source: Appl Environ Microbiol. 2026 Mar 31;92(4):e00259-26. doi: 10.1128/aem.00259-26 (PMC13101503; doi:10.1128/aem.00259-26)
Supplement: Supplemental material — Fig. S1 to S3 and Table S1. [file aem.00259-26-s0004.docx]

**SUPPLEMENTAL MATERIAL**

**Prevalence and Activity of Class II Microcins in *Serratia marcescens* Strains by Isolation Source**

Jennifer K. Parker^a^#, Leon P. Toursarkissian^a^, Joanna R. Chang^a^, Simon Sanchez-Paiva^a^, Angela C. O’Donnell^a^, Halimot O. Badmus^a^, Maria Patricia Nunez^b^, Anne-Catrin Uhlemann^c^, Bryan W. Davies^a,d^#

^a^Department of Molecular Biosciences, The University of Texas at Austin, Austin, TX, USA

^b^Department of Microbiology & Immunology, Columbia University Irving Medical Center, New York, NY, USA

^c^Division of Infectious Diseases, Columbia University Irving Medical Center, New York, NY, USA

^d^John Ring LaMontagne Center for Infectious Diseases, The University of Texas at Austin, Austin, TX, USA

**SUPPLEMENTAL FIGURES**

**
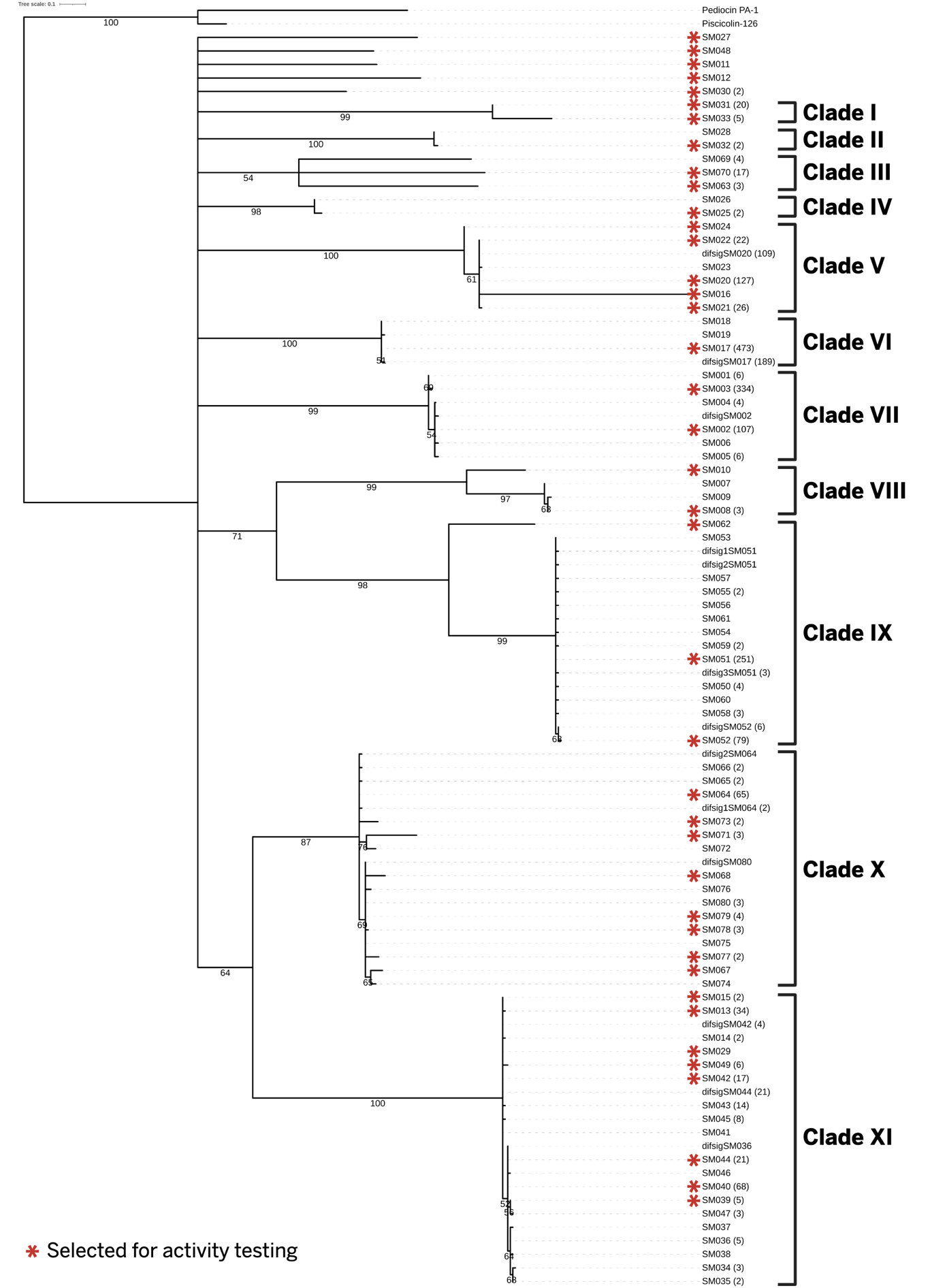
**

**Supplemental Figure 1.** Phylogeny of 93 unique putative microcins identified from *Serratia marcescens* genomes. The maximum likelihood tree was generated from an amino acid sequence alignment of the putative microcins and two Gram-positive bacteriocins (pediocin PA-1 and piscicolin-126) as the outgroup. Hits preceded by “difsig” indicates they had a different signal sequence but the same core microcin sequence as another hit with the same identification number. Hits were identified using cinful v1^1^. The total number of hits per unique sequence, if greater than one, are indicated in parentheses. Hits selected for antibacterial activity testing (*n* = 40) are indicated with an asterisk (*).

**
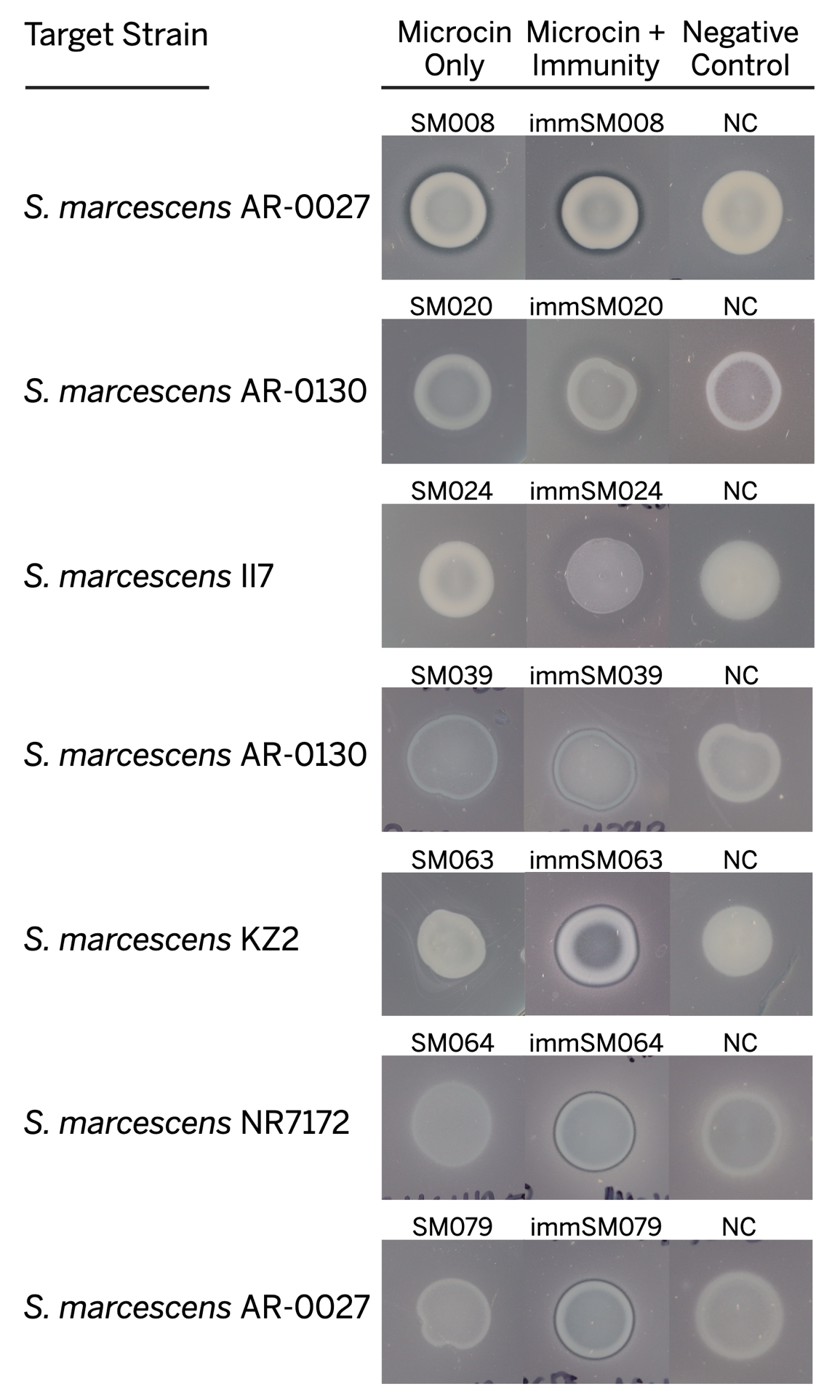
**

**Supplemental Figure 2.** *Serratia marcescens* class II microcin cognate immunity protein coexpression testing. Putative immunity proteins were tested for their effect on class II microcin zone of inhibition (ZOI) assays. Heterologous secretion of a microcin +/- immunity protein from *E. coli* spotted onto an example *S. marcescens* target strain was assayed. ZOI results are shown for the 7 class II microcins shown to be antibacterial toward one or more strains of *S. marcescens*.

**
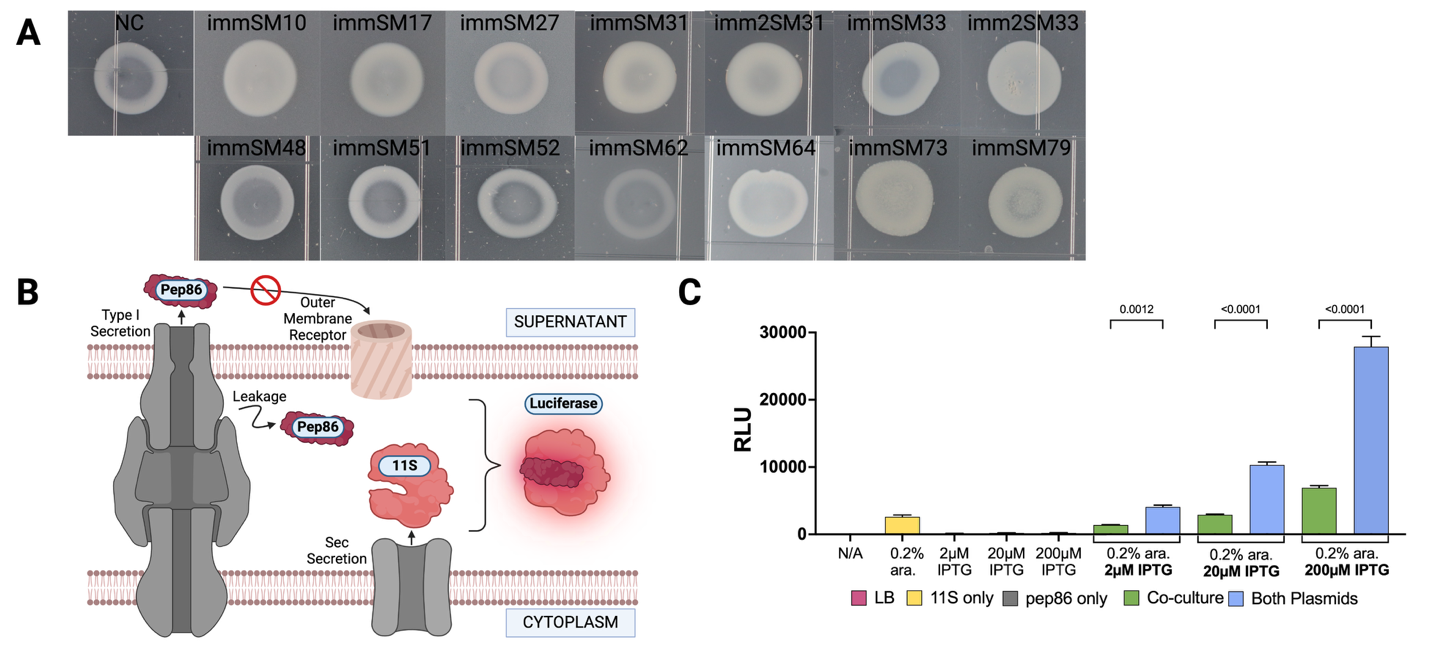
**

**Supplemental Figure 3.** *Serratia marcescens* class II microcin zone of inhibition (ZOI) testing against *E. coli*. **A)** ZOI assays of *E. coli* DH5α strains secreting *S. marcescens* microcins or the empty vector negative control (NC) spotted onto lawns of *E. coli* W3110 target cells. These microcins (*n* =12) were previously found to inhibit the growth of the *E. coli* DH5α secretor (Fig. 2A), so here they are coexpressed with their putative cognate immunity proteins. No ZOI are observed against *E. coli* W3110 for these microcins. These are representative images from triplicate assays. **B)** Split luciferase complementation assay to detect microcin secretion system leakage into the periplasm. Pep86 is secreted from the cytoplasm to the supernatant through our *E. coli* microcin type I secretion system^2^. 11S is secreted from the cytoplasm to the periplasm via the sec secretion system. If pep86 leaks into the periplasm during secretion, it can complement 11S and luminescence is detected. **C)** Microcin secretion system leakage into the periplasm. *E. coli* DH5α is transformed with plasmids for extracellular secretion of pep86 (pMMB67EH pep86-G3P2 + cvaAB) and periplasmic secretion of 11S (pBAD malE-11S). Cells are treated with arabinose to induce 11S expression and IPTG to induce pep86 expression. As IPTG concentration increases, luminescence increases, indicating pep86 in the periplasm is complementing 11S. Controls include *E. coli* DH5α containing each plasmid individually and plain LB medium. Only the ‘Both Plasmids’ treatment produces significantly increased luminescence compared to co-culture of cells with 11S and pep86 expressed individually (one-way ANOVA with post hoc multiple comparisons).

**Supplemental Table 1.** *Serratia marcescens* strains (*n* = 24) used as target strains in microcin zone of inhibition activity screening assays.

| **Strain** | **Source** | **Source Species** | **Assembly Accession** | **Reference** |
| --- | --- | --- | --- | --- |
| ATCC 13880 | Water | Pond | GCA_017654245.1 | Martinec and Kocur^3^ |
| Db11 | Insect | *Drosophila melanogaster* (fruit fly) | GCA_000513215.1 | Flyg et al. 1980^4^ |
| KZ2 | Insect | *Apis mellifera* (honey bee) | GCA_002915475.1 | Raymann et al. 2018^5^ |
| KZ11 | Insect | *Apis mellifera* (honey bee) | GCA_002915445.1 | Raymann et al. 2018^5^ |
| KZ19 | Insect | *Apis mellifera* (honey bee) | GCA_002915435.1 | Raymann et al. 2018^5^ |
| N10A28 | Insect | *Apis mellifera* (honey bee) | GCA_009834305.1 | Powell,J.E. and Moran,N.A. |
| Ss1 | Insect | *Apis mellifera* (honey bee);  *Varroa destructor* (Varroa mite) | GCA_001889685.1 | Burritt et al. 2016^6^ |
| AR-0027 | Human | *Homo sapiens* (human) | GCA_002947235.1 | CDC BIT Panel |
| AR-0121 | Human | *Homo sapiens* (human) | GCA_003071625.1 | CDC CRE Panel |
| AR-0122 | Human | *Homo sapiens* (human) | GCA_003204405.1 | CDC CRE Panel |
| AR-0123 | Human | *Homo sapiens* (human) | GCA_003071605.1 | CDC CRE Panel |
| AR-0124 | Human | *Homo sapiens* (human) | GCA_003071565.1 | CDC CRE Panel |
| AR-0130 | Human | *Homo sapiens* (human) | GCA_003071585.1 | CDC CRE Panel |
| AR-0131 | Human | *Homo sapiens* (human) | GCA_003204075.1 | CDC CRE Panel |
| IL7 | Human | *Homo sapiens* (human) | CP170117 | This publication |
| KP1028 | Human | *Homo sapiens* (human) | SAMN52858952 | This publication |
| KP1041 | Human | *Homo sapiens* (human) | SAMN52858953 | This publication |
| KP1176 | Human | *Homo sapiens* (human) | SAMN52858954 | This publication |
| NR0342 | Human | *Homo sapiens* (human) | SAMN52858955 | This publication |
| NR0621 | Human | *Homo sapiens* (human) | SAMN52858956 | This publication |
| NR0903 | Human | *Homo sapiens* (human) | SAMN52858957 | This publication |
| NR2869 | Human | *Homo sapiens* (human) | SAMN52858958 | This publication |
| NR3317 | Human | *Homo sapiens* (human) | SAMN52858959 | This publication |
| NR7172 | Human | *Homo sapiens* (human) | SAMN52858960 | This publication |

**REFERENCES**

1. Cole, T.J., Parker, J.K., Feller, A.L., Wilke, C.O., and Davies, B.W. (2022). Evidence for Widespread Class II Microcins in. Appl Environ Microbiol *88*, e0148622. 10.1128/aem.01486-22.

2. Kim, S.Y., Parker, J.K., Gonzalez-Magaldi, M., Telford, M.S., Leahy, D.J., and Davies, B.W. (2023). Export of Diverse and Bioactive Small Proteins through a Type I Secretion System. Appl Environ Microbiol *89*, e0033523. 10.1128/aem.00335-23.

3. T, M., and M, K. (1961). The taxonomic status of Serratia marcescens Bizio. International Journal of Systematic and Evolutionary Microbiology *11*, 7-12.

4. Flyg, C., Kenne, K., and Boman, H.G. (1980). Insect pathogenic properties of Serratia marcescens: phage-resistant mutants with a decreased resistance to Cecropia immunity and a decreased virulence to Drosophila. J Gen Microbiol *120*, 173-181. 10.1099/00221287-120-1-173.

5. Raymann, K., Coon, K.L., Shaffer, Z., Salisbury, S., and Moran, N.A. (2018). Pathogenicity of Serratia marcescens Strains in Honey Bees. mBio *9*. 10.1128/mBio.01649-18.

6. Burritt, N.L., Foss, N.J., Neeno-Eckwall, E.C., Church, J.O., Hilger, A.M., Hildebrand, J.A., Warshauer, D.M., Perna, N.T., and Burritt, J.B. (2016). Sepsis and Hemocyte Loss in Honey Bees (Apis mellifera) Infected with Serratia marcescens Strain Sicaria. PLoS One *11*, e0167752. 10.1371/journal.pone.0167752.
